# Investigations of Vacancy Structures Related to Their Growth in *h*-BN Sheet

**DOI:** 10.1186/s11671-017-2194-6

**Published:** 2017-07-06

**Authors:** Junga Ryou, Jinwoo Park, Suklyun Hong

**Affiliations:** Graphene Research Institute and Department of Physics, Sejong University, Seoul, 143-747 Korea

## Abstract

The atomic, electronic, and magnetic properties of vacancy structures with triangular shape related to their growth in single hexagonal boron nitride (*h*-BN) sheet are investigated using density functional theory calculations. We find that the optimized structures of triangular vacancies depend on the vacancy sizes with N-terminated zigzag edge. Then, vacancy structures obtained during the vacancy evolution in *h*-BN sheet are considered by removing a boron-nitrogen pair (BN pair) from edges of triangular vacancies. The magnetic properties of those vacancy structures are investigated by local density of states and spin densities. It is found that the stability of the optimized structures with a BN missing pair depends on the BN-pair missing position: the most stable structure is a BN-pair missing structure at the edge face region with the smallest magnetic moment.

## Background

Hexagonal boron nitride (*h*-BN) sheet is a single-layered material similar to graphene, consisting of equal numbers of boron and nitrogen atoms and it has attractive physical properties in relation to the application of nanodevices. During its synthesis, single-layer *h*-BN sheet has various defects such as vacancies and grain boundaries [1, 2]. These defects can change atomic and electronic structure of single-layer *h*-BN sheet and thus affect performance of *h*-BN-based devices.

Because *h*-BN sheet consists of two types of atoms, in contrast to graphene sheet, the edge structures of its clusters, nanoribbons, or nanoholes divide into two types: N-terminated and B-terminated. The most stable structure of the edge of cluster has N-terminated edge with zigzag structure [3, 4]. In previous theoretical studies, the atomic and electronic structures of vacancy structures in single-layer *h*-BN sheet depend on the type of termination atoms and their vacancy size [3–11]. That is, the calculated stability of triangle vacancy structures and the magnetic properties were found to depend on the type of terminated atoms and vacancy sizes of triangle vacancy due to lone electrons at edge atoms. The triangular vacancy structures were found in experiments for using a free-standing *h*-BN sheet [12–15]. Electron beam irradiation results in increasing size of vacancy structures that maintain triangular shape [12, 13] regardless of the vacancy size.

Recently, we reported the study for growth of triangular vacancy of single-layer *h*-BN sheet [15]. It was observed in the experiment that atoms in *h*-BN sheet are ejected in the form of the bundles, not each atom, at the edge of vacancy structures. Furthermore, we briefly mentioned theoretical results to explain the growth of vacancy in *h*-BN sheet with triangular shape.

In this paper, we address the detailed study of the atomic structures of triangular vacancy of single-layer *h*-BN sheet. The locally stable structures of triangular vacancies are found to depend on the vacancy sizes with N-terminated zigzag edge. Then, by increasing the vacancy size, we investigate the stability of the optimized structures with a BN missing pair and their magnetic properties.

## Computational methods

We have performed the density functional theory calculations using the Vienna ab initio simulation package (VASP) [16, 17]. The plane-wave basis set with the energy cutoff of 400 eV is employed to describe electronic wave functions. The ions are represented by projector-augmented wave potentials [18, 19] and generalized gradient approximation is employed to describe the exchange-correlation functional [20, 21]. To take the weak van der Waals (vdW) interactions, we adopt Grimme’s DFT-D2 vdW correction [22] based on a semi-empirical GGA-type theory.

The atomic positions of all structures are relaxed with residual forces smaller than 0.01 eV/Å. For the Brillouin-zone integration, we use only gamma point in the Monkhorst-Pack special k-points scheme. The lattice constant of our model is calculated to be 2.56 Å, which is in agreement with experimental value [23]. To study the difference in the reconstructed structures after BN-pair missing, we consider (9 × 9) and (15 × 15) supercell in our calculations.

## Results and Discussion

### Triangular Vacancy in *h*-BN Sheet

First, we have considered several vacancy sizes of single *h*-BN sheet to study the size effect of vacancy structures. Because N-terminated vacancy structure of *h*-BN sheet is more stable structure than B-terminated one [3, 4], we mainly focus on N-terminated triangular vacancy structures. To control vacancy sizes of *h*-BN sheet, we increase the number of ejected atoms in *h*-BN sheet maintaining the triangular shape. The B-terminated vacancy structures after relaxation result in small distortion in their vertex region with weakly binding between B atoms (not shown here) while the N-terminated structures shows a distinct change at the vertices of their triangular vacancy. Among different vacancy sizes of N-terminated triangular shape, we find two types of optimized (i.e., locally stable) structures. One is a symmetric structure (denoted as N-symm) in which no noticeable change of structure at vertex of triangular vacancy is found when compared with the pristine *h*-BN sheet, whereas the other is a distortion structure (denoted as NN-bond) that shows N-N bonds at all vertices of triangular hole vacancy in *h*-BN sheet.

In the cases of B monovacancy (V_1B_) in *h*-BN sheet, the optimized structure shows only one configuration that is the N-symm structure. Due to strong repulsive force between N atoms located at the vertex of triangular vacancy, the distance between N atoms increases (2.66 Å) compared to that of pristine *h*-BN sheet (2.48 Å) and B-N bond lengths at the edge of the triangular vacancy decrease.

**Fig. 1:**
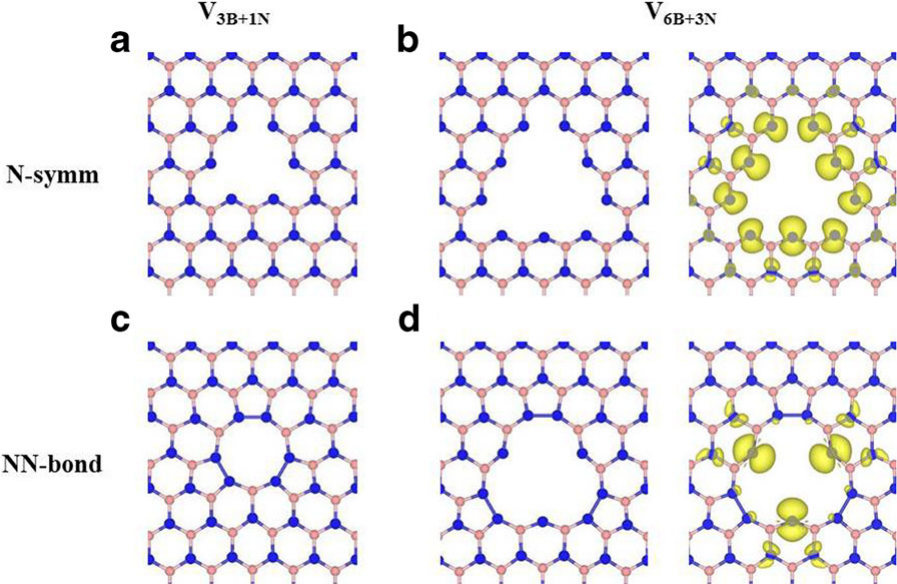
Optimized vacancy structures of **a** V_3B+1N_ and **b** V_6B+3N_ with N-symm structure and **c** V_3B+1N_ and **d** V_6B+3N_ with NN-bond structure. *Blue* and *pink balls* represent B and N atoms, respectively. Beside plots **b** and **d** are the differences in the spin density projected on the plane of the V_6B+3N_ structures

When the size of triangular vacancy of *h*-BN sheet is increases to give V_3B+1N_ and V_6B+3N_ structures, where V_*m*B+*n*N_ represents a triangular vacancy with *m* missing B atoms and *n* missing N atoms, the optimized structures can have both N-symm and NN-bond structures, as shown in Fig. 1. These results agree with the previous theoretical study for the vacancy structures [6].

The optimized structure of larger vacancy sizes than that of V_6B+3N_ structure represents only one configuration, i.e., an NN-bond structure. This large vacancy structure has the longer edge length of triangular shape than that of small vacancy structure, which means that the B-N bonds around the vacancy hole is less affected by the formation of N-N bond at vertex of vacancy hole in large vacancy structures (and thus the bond lengths between B and N atoms remain almost the same at the edge of vacancy structure). Calculated bond lengths between N atoms at the vertices of triangular vacancies and relative energies of two types of vacancy structures are given in Table 1. We find that the N-N bond lengths and relative energies depend on the size of vacancies. The difference in relative energies between N-symm and NN-bond structures decreases with increasing size of triangular vacancy structure. In contrast, the B-terminated vacancy structures turn out to be only one structure with a weak B-B bond at the vertex regardless of their sizes (see Table 1).

**Table 1:** The distances d_0_ (Å) between N atoms (N-terminated vacancy) or B atoms (B-terminated vacancy) at vertices of vacancy structures, calculated total magnetic moments *M* (*μ*_*B*_), and the relative energies E_r_ (eV) for triangular vacancy configurations of *h*-BN sheet

N-terminated vacancy								
	V_1B_	V_3B+1N_		V_6B+3N_		V_10B+6N_	V_15B+10N_	V_21B+15N_
	N-symm	N-symm	NN-bond	N-symm	NN-bond	NN-bond	NN-bond	NN-bond
d_0_ (Å)	2.66	2.49	1.65	2.48	1.67	1.67	1.67	1.67
E_r_ (eV)	-	1.74	0.00	1.12	0.00	-	-	-
*M* (*μ*_*B*_)	3	6	0	9	3	6	9	12
B-terminated vacancy								
	V_1N_		V_1B+3N_		V_3B+6N_		V_6B+10N_	
d_0_ (Å)	2.28		1.96		1.97		1.98	
*M* (*μ*_*B*_)	3		0		3		6	

The calculated total magnetic moments of vacancy structures vary depending on the vacancy size, terminated atoms, and optimized structures (see Table 1). In the N-symm structures, the value of the magnetic moment in units of *μ*_*B*_ is equal to the number of nitrogen atoms located at the edge of triangular vacancy structures because these N atoms have dangling bonds after missing of atoms and breaking of B-N bonds in the *h*-BN sheet. However, total magnetic moments of N-N bond structures with various vacancy sizes are calculated to be different from those of N-symm structures due to formation of the N-N bonds (homopolar sigma bond) at vertices of triangular vacancy structures. The total magnetic moments for the V_3B+1N_, V_6B+3N_, and V_10B+6N_ structures with N-N bonds at vertex of vacancy are 0, 3, and 6 *μ*_*B*,_ respectively. Figure 1b, d shows the difference in spin densities for the V_6B+3N_ structures with N-symm (*M* = 9 *μ*_*B*_) and N-N bond (*M* = 3*μ*_*B*_) structures, respectively.

### BN Pair Missing at the Edge Region of Vacancy Hole

Next, we have investigated the BN-pair missing situation in N-terminated vacancy structures in detail because the size of vacancy hole structures was observed to be expanded through missing of B and N atoms at the edge of triangular vacancy structures in the experiment [14]. It was also reported that when the vacancies grow maintaining triangular shape in *h*-BN sheet, B and N atoms are ejected preferentially with pairs or bundles from the edge face of vacancy structures [15].

**Fig. 2:**
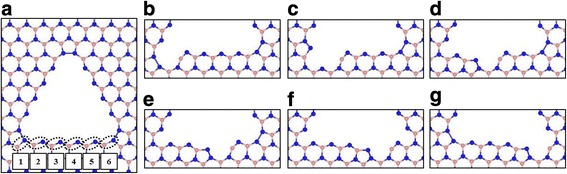
Optimized structures of **a** V_21B+15N_ vacancy structure with possible missing positions of BN-pair and **b**–**g** V_22B+16N_ vacancy structures after a BN-pair missing at specific positions. The *dotted circles* with numbering in **a** represent possible positions of BN-pair missing. The positions with numbering 1 to 6 is denoted as **b** corner-1, **c** face-1, **d** face-2, **e** face-3, **f** face-4, and **g** corner-2 missing structures, respectively

To study stability of vacancy structures depending on the missing position, we increase the supercell size of *h*-BN sheet up to 15 × 15 unit cell and obtain the larger vacancy size such as V_15B+10N_ and V_21B+15N_. It is found that the optimized relaxations for those vacancies result in only one stable atomic configuration, i.e., the NN-bond configuration. The N-N bond lengths at the vertices and total magnetic moments are shown in Table 1. We select a large N-terminated V_21B+15N_ triangular vacancy structure embedded in the supercell to consider more missing positions (Fig. 2a). As shown in the Fig. 2a, the number of possible positions of BN-pair missing at the edge of V_21B+15N_ vacancy structure is six. After relaxation of the vacancy structure with BN-pair missing at different positions, we find the difference in the optimized structures depending on the missing positions as shown in the Fig. 2b–g. The optimized structures are divided into three types depending on the missing positions; corner missing (1 and 6), near corner missing (2 and 5), and middle missing (3 and 4) positions.

After one BN-pair missing at the edge of triangular vacancy structure, the optimized structure shows reconstructed BN hexagonal open ring near the missing position in which the B-N bond lengths at the distorted BN ring are slightly shorter; this means that the interactions between B and N atoms become stronger and change the arrangement of the electron charge distribution in the B-N bonds. The corner-1 missing structure (missing numbering 1) is almost unchanged except the region of distorted BN open ring as shown in the Fig. 2b.

It is found that other structures (missing numbering 2–6) have one N dimer with pentagonal shape at the edge, shown in Fig. 2d–g, except Fig. 2c with N dimer located at the vertex. That is, the N atoms near the missing position have the dangling bond due to the missing and form the N dimer (see Fig. 2d–g). The presence of the N dimer in each structure influences its stability and magnetic properties. We calculate the relative energies and total magnetic moments of BN-pair missing structures obtained from V_21B+15N_ vacancy structure, which are listed in Table 2.

**Table 2:** The relative energies E_r_ (eV) and total magnetic moments *M* (*μ*_*B*_) of optimized configurations with a BN-pair missing at specific positions

Missing number	1 corner-1	2 face-1	3 face-2	4 face-3	5 face-4	6 corner-2
E_r_ (eV)	3.90	0.68	0.33	0.00	0.52	2.18
*M* (*μ*_*B*_)	12	10	10	8	10	12

**Fig. 3:**
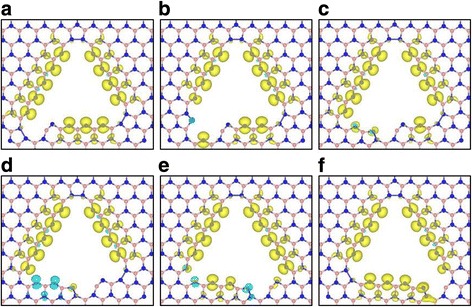
The spin density (ρ_spin up_ -ρ_spin down_) distributions for the optimized structures of BN-pair missing. *Yellow* and *light blue* isosurfaces indicate the positive and negative values of spin densities, respectively

Based on the relative energies, we find that the stability of the BN-pair missing structures increases when the missing position gets closer to the center of the triangle edge (see Table 2). Calculated total magnetic moments caused by terminated N atoms at the edge of optimized vacancy structures depend on the missing position. The magnetic moments of two corner missing structures are the same (*M* = 12*μ*_*B*_). After the missing, the number of the terminated N atoms is 13 in the corner missing structures, which might give the magnetic moment *M* = 13*μ*_*B*_. However, the magnetic moment of an N atom in the distorted BN open rings vanishes due to the rearrangement of charge distribution as mentioned above. The magnetic moments of other structures vary depending on the missing positions due to the presence of reconstructed BN open ring and/or N dimer located near the missing point. Figure 3 shows the spin densities of optimized structures obtained after BN-pair missing. From these spin densities, we know where the magnetic moments listed in Table 2 come from.

**Fig. 4:**
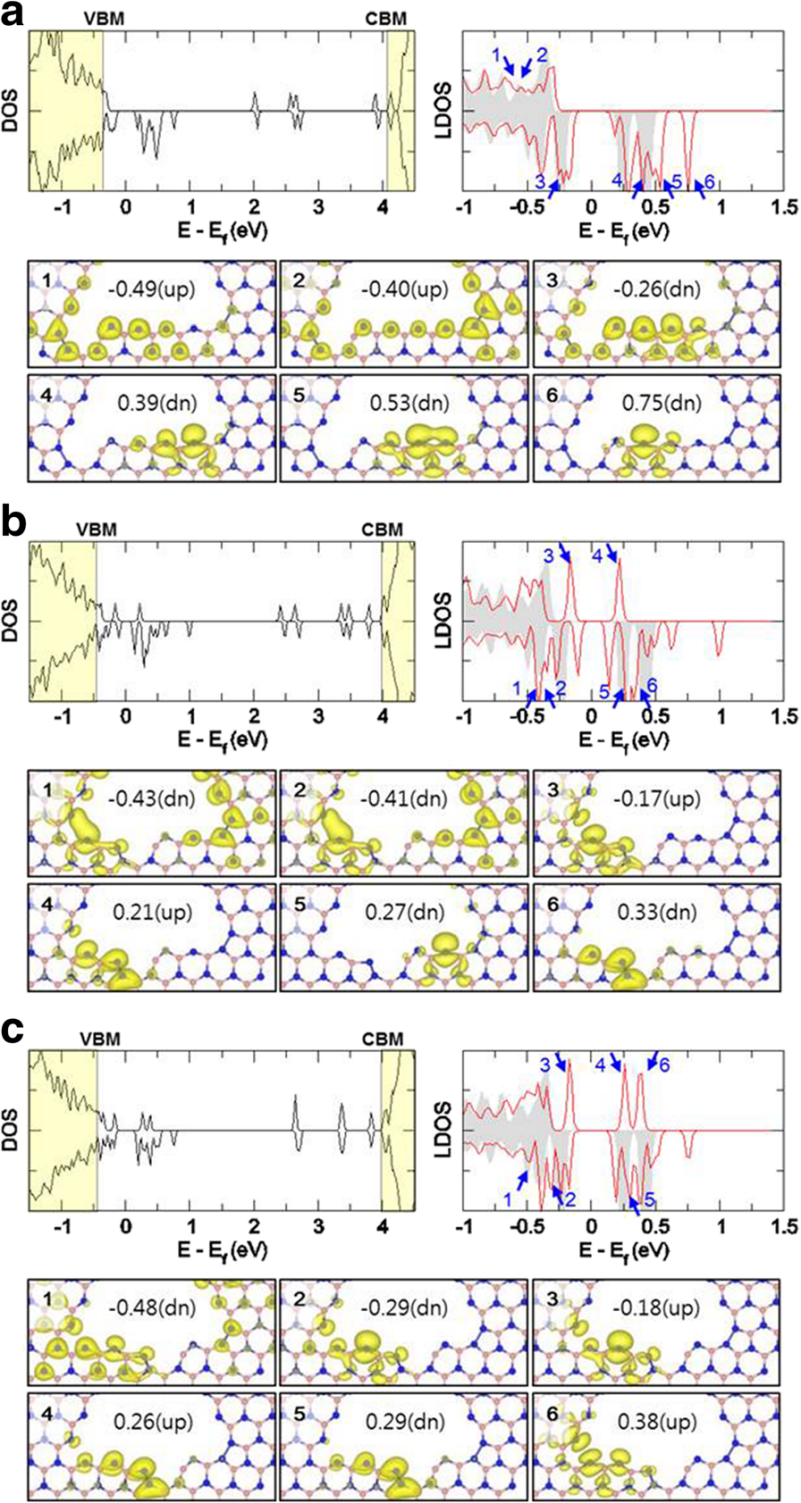
Total DOS, LDOS of edge N atoms and the spin density plots near the Fermi level for the optimized structures of V_21B+15N_ vacancy structure with BN-pair missing: **a** corner-1 missing, **b** face-2 missing, and **c** face-3 missing structures. The light *yellow* shaded regions in total DOS represent the regions of valence bands and conduction bands of the pristine *h*-BN sheet, respectively. The *grey* shaded and red solid-line features are LDOS of N atoms before and after BN-pair missing at the edge of *triangular* vacancy structure

For accurate analysis of differences between optimized structures depending on the missing positions, we select three configurations (corner-1, face-2, and face-3) among six BN-pair missing structures and calculate their electronic density of state (DOS). In the DOS plots, the defect states are located inside the band gap of the pristine *h*-BN sheet, as shown Fig. 4, where the valence band maximum and conduction band minimum of the pristine *h*-BN sheet are indicated by VBM and CBM, respectively. In the local DOS (LDOS) plot, the grey shaded regions and the red solid lines indicate LDOS of N atoms of the vacancy structures before and after BN-pair missing, respectively. Especially, the states of edge N atoms are concentrated around the Fermi level in the LDOS plot. As shown in the DOS and LDOS plots, the spin states of edge N atoms show the asymmetrical features. The corner-1 missing structure in Fig. 4a shows dangling bond states of N atoms in the range of −0.5 to 1.0 eV of the DOS and LDOS plots: noticeably, the dangling bond states localized only in the edge face region come mostly from spin-down states of LDOS (see spin density plots related to peak positions numbered 3 to 6 in LDOS plot). In the LDOS plots of two face-missing structures (Fig. 4b, c), not only the spin-down states but the spin-up states of edge N atoms also appear as the dangling bond states localized only in the edge face region near the Fermi level (−0.5~1.0 eV). That is, these spin-up and spin-down plots are related to peak positions numbered 3 to 6 in the LDOS plot of Fig. 4b and those numbered 2 to 5 in the LDOS plot of Fig. 4c. On the other hand, all the BN-pair missing structures have the energy band gap. The band gaps are about 0.35, 0.24, and 0.36 eV for the corner-1, face-2, and face-3 missing structures, respectively.

## Conclusions

We have investigated the structural and electronic properties of the triangular vacancy structures of *h*-BN sheet using first-principles calculations. The optimized triangular vacancy structures were found to depend on their vacancy size. The most stable configuration of large vacancy structures has the N-N bond at each vertex of triangular vacancy, which determines its magnetic moments. When the missing of a BN pair occurs at the edge of triangular vacancy structure with a large hole size in the *h*-BN sheet, as observed in the experiment, the most stable structure is found to be a face missing structure with formation of N-N bonds. The magnetic moments and LDOS of the optimized structures depend on the missing positions of BN-pair at the edge of triangular vacancy.
